# Sleeping, Smoking, and Kidney Diseases: Evidence From the NHANES 2017–2018

**DOI:** 10.3389/fmed.2021.745006

**Published:** 2021-09-28

**Authors:** Chia-Chao Wu, Han-En Wang, Yi-Chun Liu, Cai-Mei Zheng, Pauling Chu, Kuo-Cheng Lu, Chi-Ming Chu, Yu-Tien Chang

**Affiliations:** ^1^Division of Nephrology, Department of Internal Medicine, National Defense Medical Center, Tri-Service General Hospital, Taipei, Taiwan; ^2^National Defense Medical Center, Department and Graduate Institute of Microbiology and Immunology, Taipei, Taiwan; ^3^School of Public Health, National Defense Medical Center, Taipei City, Taiwan; ^4^Division of Nephrology, Department of Internal Medicine, Shuang Ho Hospital, Taipei Medical University, New Taipei City, Taiwan; ^5^TMU Research Centre of Urology and Kidney, Taipei Medical University, Taipei, Taiwan; ^6^Department of Internal Medicine, School of Medicine, College of Medicine, Taipei Medical University, Taipei, Taiwan; ^7^Division of Nephrology, Department of Medicine, School of Medicine, Fu-Jen Catholic Hospital, Fu-Jen Catholic University, New Taipei City, Taiwan; ^8^Department of Surgery, National Defense Medical Center, Songshan Branch of Tri-Service General Hospital, Taipei City, Taiwan; ^9^Division of Biostatistics and Informatics, Department of Epidemiology, National Defense Medical Center, School of Public Health, Taipei, Taiwan; ^10^Department of Public Health, China Medical University, Taichung City, Taiwan; ^11^Department of Public Health, Kaohsiung Medical University, Kaohsiung City, Taiwan

**Keywords:** sleep, smoking, kidney function, NHANES, eGFR

## Abstract

**Study Objectives:** Smoking and sleep are modifiable factors associated with the chronic kidney diseases. However, the interaction of smoking and sleep on the renal function are still unclear. Therefore, we aimed to evaluate the interactive impacts of smoking and sleep on the renal function.

**Methods:** Data were obtained from the National Health and Nutrition Examination Survey. The study population were categorized into nine subgroups by smoking (smoking every day, sometimes, and non-smokers recently) and sleep duration (short duration ≤ 6 h, normal duration 6–9 h, and longer duration ≥ 9 h on the weekdays).

**Results:** The study group with a short sleep duration had significantly higher serum cotinine and hydrocotinine levels compared with the other two sleep groups. After adjusting the demographic characteristics (age, race, body mass index, and marital status), sleep quality (snoring or breathing cessation), and comorbidities (diabetes mellitus, hypertension, high cholesterol, anemia, congestive heart failure, coronary heart disease, and stroke), non-smokers with short or long sleep duration had significant lower estimated glomerular filtration rate (eGFR) levels than the study group who smoked every day and slept ≤ 6 h. The effects of sleep duration on eGFR levels varied with smoking status. For the study group smoking every day, eGFR levels increased as sleep duration decreased, whereas for the study group smoking sometimes, eGFR levels increased as sleep duration increased. The U-shaped effects of eGFR levels were observed among non-smokers whose normal sleep duration was associated with better eGFR levels. Normal sleep duration was an important protective factor of the renal function for non-smokers than smokers.

**Conclusions:** The effects of sleep duration on eGFR levels varied with smoking status. Normal sleep duration was a protective factor and more crucial for non-smokers than for smokers.

## Introduction

Chronic kidney diseases (CKDs) represent a heavy burden on the healthcare system because of the increasing number of patients, high risk of progression to end-stage renal disease, and poor prognosis with respect to morbidity and mortality (1). Sleep and smoking are two main modifiable factors of CKDs (2). Sleep plays an important role in every aspect of physiology. Sleep reduction has become highly prevalent owing to access to artificial indoor lighting, smartphones, and daily living activities. A population-based study showed that 22.3% of men and 28.9% of women aged ≥ 16 years told their doctors that they had trouble sleeping (3). Short sleep and long sleep duration (4) as well as poor objective sleep quality have been shown to be associated with the lower estimated glomerular filtration rate (eGFR) and CKD development (5–7).

Smoking is a leading cause of preventable deaths worldwide (8), and increases the risk of developing CKDs (9). The association of longer smoking duration with a higher risk of progression of CKDs was evident particularly in patients with eGFR <45 ml/min/1.73 m^2^ and proteinuria ≥ 1.0 g/g. By contrast, the risk of adverse kidney outcomes decreased with longer smoking-free periods among former smokers (10).

Smoking and sleep problems have been demonstrated to have a reciprocal relationship with each other (10, 11). A strong relationship between smoking and subsequent sleep problems was observed in adolescents; this relationship was independent of demographics, snoring, or sleep apnea (SA), body mass index (BMI), depressive symptoms, alcohol use, and soda consumption (12). However, to the best of our knowledge, no study has evaluated the interaction effect of smoking and sleep duration on kidney function. Therefore, we aimed to evaluate the interactive impacts of smoking and sleep on renal function using datasets from the National Health and Nutrition Examination Survey (NHANES).

## Methods

### Data Source

The National Health and Nutrition Examination Survey (13) is a program of studies designed to assess the health and nutritional status of adults and children in the United States. All the participants provided informed written consent for the study, which was approved by the Ethics Review Board of the National Center for Health Statistics. We used the NHANES datasets from 2017 to 2018, including all the cases of demographic variables (DEMO_J), questionnaire data of smoking and cigarette use (SMQ_J), sleep disorders (SLQ_J), laboratory data of albumin and creatinine—urine (ALB_CR_J), cotinine and hydroxycotinine—serum (COT_J), standard biochemistry profile (BIOPRO_J), blood pressure and cholesterol (BPQ_J), diabetes (DIQ_J), and medical conditions (MCQ_J).

### Chronic Kidney Disease Epidemiology Collaboration Equations for eGFR

The R package of “CKDEpi.creat” (14) and parameters of serum creatinine, sex, age, and ethnicity were used to calculate eGFR using the CKD-EPI equation. The CKD-EPI equation is expressed as a single equation as follows: GFR = 141 × min (S_Cr_/κ, 1)^α^ × max (S_Cr_/κ, 1)^−1.209^ × 0.993 ^age^ ×1.018 [if female] × 1.159 [if black], where S_Cr_ is the standardized serum creatinine in mg/dl, κ is 0.7 for women and 0.9 for men, a is −0.329 for women and −0.411 for men, min indicates the minimum of S_Cr_/κ or 1, and max indicates the maximum of S_Cr_/κ or 1 (15). eGFR values are presented in ml/min/1.73 m^2^.

### Statistical Analysis

Statistical analysis was performed using R version 4.0.2 (16). The testing index distribution was skewed; hence, we used the R package “bestNormalize” (17) to normalize the data. The testing index of blood urea nitrogen (mmol/l) was normalized using the center scale transformation whereas that of uric acid (μmol/l) was normalized using square root transformation. The levels of serum creatinine (μmol/l), urine creatinine (μmol/l), albumin–creatinine ratio (mg/g), urine albumin (μg/ml), serum cotinine (ng/ml), and serum hydroxycotinine (ng/ml) were normalized using log transformation. The descriptive statistics of the testing index are presented as non-normalized figures for clinical use. Transformed figures were used in the multivariable linear regression models. We performed univariable and multivariable linear regression analyses to determine whether sleep and smoking are associated with renal function while controlling for demographic characteristics (age, gender, and marriage), body measurement, marital status, sleep quality, and comorbidities. Variables that were significant in the univariable models were included in the multivariable analyses. The study population was divided into nine subgroups based on sleep duration on weekdays ( ≤ 6, 6–9, and ≥ 9 h) and smoking (smoking every day, smoking sometimes, and never smoking recently) for sensitivity analysis. Forest plots were used to present the difference in eGFR levels among the nine subgroups.

## Results

### Risk Factors of Kidney Diseases

The significant risk factors of kidney diseases were as follows: (1) demographic characteristics: male, older age, non-Hispanic white race, higher BMI and widowed/divorced or separated status; (2) sleep quality: frequent snoring or breath cession; (3) smoking: higher serum cotinine and hydrocotinine levels, older age at the start of smoking cigarettes regularly, no smoking recently, smoking since waking for 6–30 min, higher number of smoking days, number of cigarettes in the past 30 days; (4) comorbidities: hypertension, high cholesterol levels, diabetes mellitus, failing kidneys, anemia, congestive heart failure, coronary heart disease, stroke, chronic obstructive pulmonary disease, and malignancy. Blood urea nitrogen, serum creatinine, uric acid, and the albumin–creatinine ratio were negatively associated with eGFR levels (Table 1).

**Table 1:** Univariable linear regression of the renal function.

	**eGFR (ml/min/1.73 m** ^**2**^ **)**	
	**B**	***p*-values**
**Demographic characteristics**		
**Female (ref: male)**	2.64	^***^
**Age (year)**	−1.07	^***^
**Race (ref: Mexican American)**		
Other Hispanic	−6.86	^***^
Non-Hispanic white	−15.36	^***^
Non-Hispanic black	−4.24	^**^
Other Race—including multi-racial	−5.43	^***^
**Weight (kg)**	−0.21	^***^
**Height (cm)**	−0.20	^***^
**BMI (kg/m** ^**2**^ **)**	−0.64	^***^
**Marital status (ref: Married or living with partner)**		
Never married	12.55	^***^
Widowed, divorced, or separated	−11.51	^***^
**Renal function**		
Blood urea nitrogen (mmol/L)	−7.76	^***^
Creatinine, refrigerated serum (umol/L)	−0.40	^***^
Uric acid (umol/L)	−0.10	^***^
Albumin creatinine ratio (mg/g)	−0.01	^***^
**Metabolites of nicotine**		
Serum cotinine (ng/mL)	−0.02	^***^
Serum hydrocotinine (ng/mL)	−0.05	^***^
**Sleep quality**		
**Sleep duration on weekdays (h)**	−0.11	0.60
**How often do you snore? (ref: never)**		
Rarely−1–2 nights a week	−2.51	^*^
Occasionally−3–4 nights a week	−7.91	^***^
Frequently−5 or more nights a week	−8.60	^***^
**How often do you snort or stop breathing (ref: never)**	
Rarely−1–2 nights a week	−2.66	^*^
Occasionally−3–4 nights a week	−6.78	^***^
Frequently−5 or more nights a week	−10.90	^***^
**Smoking status**		
**Age started smoking cigarettes regularly**	−0.23	^**^
**Do you now smoke cigarettes? (ref: Every day)**		
Some days	0.93	0.63
Not at all	−12.41	^***^
**How soon after waking do you smoke (ref: Within 5 min)**		
6 ~ 30 min	−4.95	^*^
≥30 min	−1.81	0.40
**# days smoked cigarettes during past 30 days**	−0.42	^***^
**Average # cigarettes/day during past 30 days**	−0.21	^*^
**Tried to quit smoking**	0.78	0.60
**Comorbidities**		
**High blood pressure**	−21.40	^***^
**High cholesterol**	−17.85	^***^
**Diabetes (ref: Yes)**		
No	22.64	^***^
Borderline	10.27	^***^
**Failing kidneys**	−37.57	^***^
**Anemia**	−7.14	^***^
**Congestive heart failure**	−28.80	^***^
**Coronary heart disease**	−23.38	^***^
**Stroke**	−21.78	^***^
**Chronic obstructive pulmonary disease**	−16.74	^***^
**Malignancy**	−20.38	^***^

* *p < 0.05*,

** *p < 0.01*,

*** *p < 0.001. Ref, reference group*.

### Baseline Characteristics and the Impacts of Sleep Duration on the Renal Functions

The normal sleep duration group (nmSleep) was the youngest (33.2 ± 24.3 years) and had the lowest BMI on average (25.6 ± 8.14 kg/m^2^). The primary marital status of nmSleep was married or living with partners (60.5%) compared with the other two sleep groups with less or more sleep duration (abbreviation: lessSleep and moreSleep). Except for malignancy, nmSleep had a lower prevalence of hypertension, high cholesterol, DM, failing kidneys, anemia, congestive heart failure, coronary heart disease, heart attack, stroke, and COPD than the other two sleep groups (Table 2).

**Table 2:** Baseline characteristics of study population grouped by sleep duration.

	**Sleep duration on weekdays**			
	**≤6 h**	**6–9 h**	**≥9 h**	***p*-values**
	**(*n* = 1,118)**	**(*n* = 5,652)**	**(*n* = 1,499)**	
**Demographic characteristics**				
**Female**	512 (45.8%)	2,874 (50.8%)	841 (56.1%)	^***^
**Age (year)**	48.5 (17.8)	33.3 (24.3)	49.1 (22.0)	^***^
**Race**				^***^
Mexican American	136 (12.2%)	848 (15.0%)	223 (14.9%)	
Other Hispanic	105 (9.39%)	489 (8.65%)	140 (9.34%)	
Non-Hispanic white	314 (28.1%)	1,907 (33.7%)	538 (35.9%)	
Non-Hispanic black	367 (32.8%)	1,211 (21.4%)	335 (22.3%)	
Other Race—including multi-racial	196 (17.5%)	1,197 (21.2%)	263 (17.5%)	
**Weight (kg)**	85.2 (24.9)	65.3 (30.4)	79.3 (22.2)	^***^
**Height (cm)**	167 (10.3)	155 (22.0)	165 (9.90)	^***^
**BMI (kg/m** ^**2**^ **)**	30.3 (8.10)	25.6 (8.14)	28.9 (7.29)	^***^
**Marital status**				^**^
Married or living with partner	602 (57.6%)	1,949 (60.5%)	701 (54.1%)	
Never married	199 (19.0%)	569 (17.7%)	238 (18.4%)	
Widowed, divorced, or separated	245 (23.4%)	704 (21.8%)	356 (27.5%)	
**Comorbidities**				
**High blood pressure**	424 (37.9%)	1,181 (33.4%)	532 (35.6%)	^*^
**High cholesterol**	356 (32.2%)	1,117 (31.7%)	495 (33.2%)	0.60
**Diabetes**				^***^
Yes	154 (13.8%)	487 (8.62%)	252 (16.8%)	
No	926 (82.9%)	5,050 (89.4%)	1,212 (80.9%)	
Borderline	37 (3.31%)	113 (2.00%)	34 (2.27%)	
**Failing kidneys**	50 (4.79%)	100 (3.10%)	73 (5.64%)	^***^
**Anemia**	54 (4.84%)	177 (3.13%)	89 (5.97%)	^***^
**Congestive heart failure**	55 (5.27%)	85 (2.64%)	61 (4.72%)	^***^
**Coronary heart disease**	60 (5.74%)	121 (3.76%)	84 (6.49%)	^***^
**Stroke**	57 (5.47%)	118 (3.67%)	98 (7.56%)	^***^
**Chronic obstructive pulmonary disease**	71 (6.81%)	135 (4.19%)	87 (6.72%)	^***^
**Malignancy**	91 (8.71%)	334 (10.4%)	163 (12.6%)	^***^
**Renal function**				
Blood Urea Nitrogen (mmol/L)	5.36 (2.28)	5.14 (2.04)	5.33 (2.26)	^**^
Creatinine, refrigerated serum (umol/L)	82.6 (53.5)	75.1 (33.3)	79.6 (42.1)	^***^
eGFR (ml/min/1.73 m^2^)	95.8 (25.5)	103 (28.8)	95.0 (28.6)	^***^
Uric acid (umol/L)	330 (87.8)	320 (87.9)	319 (88.4)	^**^
Albumin creatinine ratio (mg/g)	44.0 (223)	38.1 (294)	59.9 (365)	0.06
**Metabolites of nicotine**				
Serum cotinine (ng/mL)	75.6 (152)	30.4 (95.9)	49.7 (122)	^***^
Seum hydrocotinine (ng/mL)	30.9 (82.0)	11.7 (41.6)	21.4 (58.8)	^***^

* *p < 0.05*,

** *p < 0.01*,

*** *p < 0.001 using statistical methods of ANOVA and Chi-squared test*.

**Figure 1:**
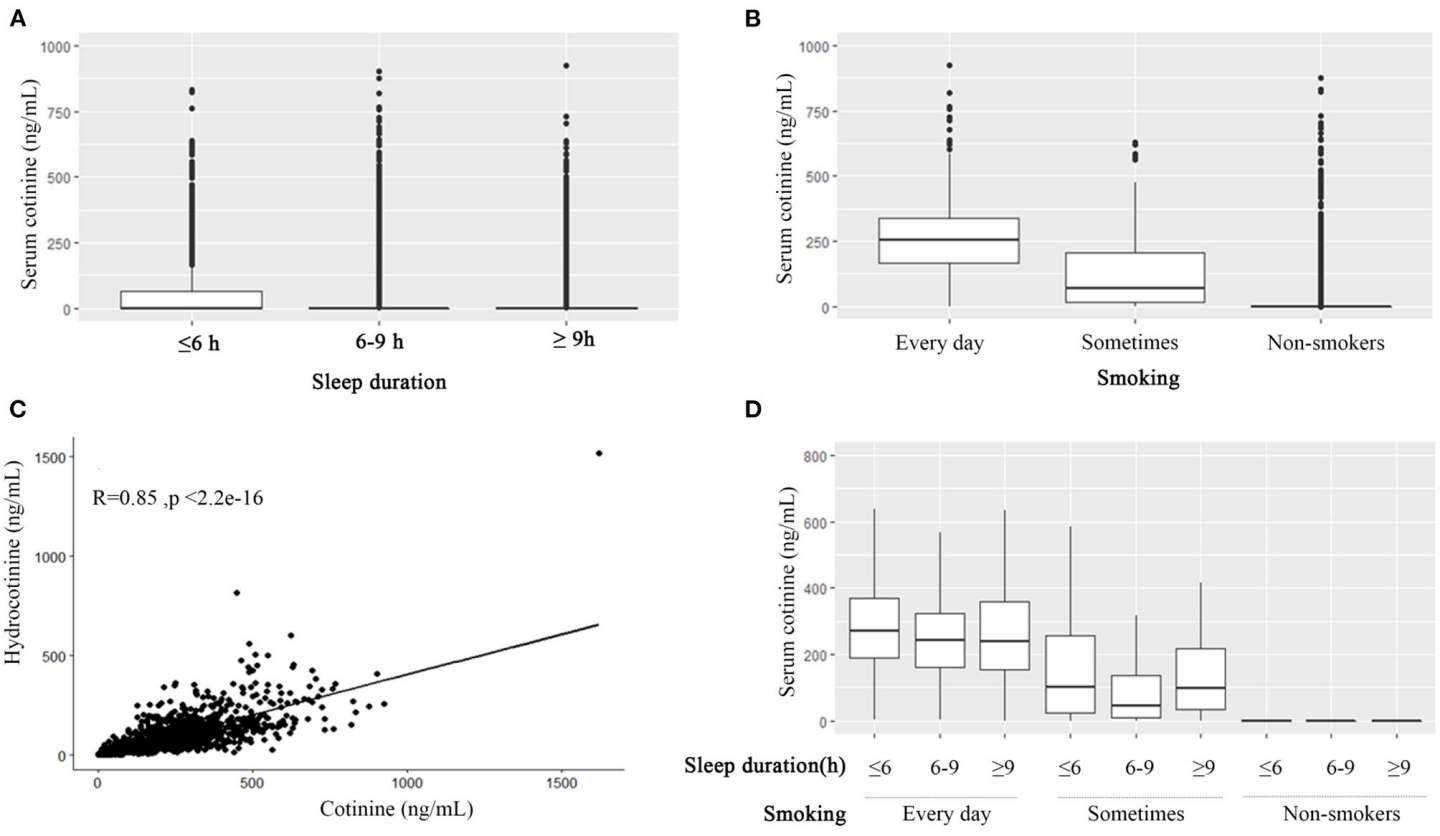
The association among smoking, cotinine, hydrocotinine, and sleep duration. Boxplots of serum cotinine levels grouped by sleep duration (≤ 6, 6–9, and ≥9 h) **(A)**, grouped by smoking frequency **(B)**, and grouped by sleep duration and smoking frequency **(D)**. **(C)**The scatter plot of cotinine and hydrocotinine with the Pearson correlation r = 0.85.

Renal function was associated with sleep duration. nmSleep and moreSleep had higher eGFR levels. nmSleep had the lowest blood urea nitrogen, the lowest serum creatinine, and a lower uric acid among the three sleep groups. No difference was found in the albumin–creatinine ratio. Taken together, nmSleep had the best renal function. The U-shaped effects of the renal function levels were observed among the sleep duration groups (Table 2). Significantly higher cotinine (75.6 ± 152 ng/ml, *p* < 0.001) and hydrocotinine (30.9 ± 82 ng/ml, *p* < 0.001) levels were observed in lessSleep compared with the other two sleep groups (Table 2; Figure 1A).

### Baseline Characteristics and the Impacts of Smoking on the Renal Functions

The non-smoker group (noSmoking) was predominantly male (63.5%), non-Hispanic White (45.7%), married or living with partners, and older (58.8 ± 17 years) and had higher BMI (30.8 ± 7.39 kg/m^2^). The prevalence of hypertension, high cholesterol, DM, failing kidneys, anemia, congestive heart failure, coronary heart disease, and malignancy was higher in noSmoking than other smoking groups of smoking every day (edSmoking) or smoking sometimes (stSmoking). The COPD prevalence was the highest in edSmoking (Table 3).

**Table 3:** Baseline characteristics of study population grouped by smoking.

	**Do you now smoking?**			
	**Every day**	**Some days**	**Not at all**	***p*-values**
	**(*n* = 805)**	**(*n* = 216)**	**(*n* = 1,338)**	
**Demographic characteristics**				
**Female**	336 (41.7%)	89 (41.2%)	488 (36.5%)	^*^
**Age (year)**	47.7 (16.0)	46.2 (16.1)	58.8 (17.0)	^***^
**Race**				^***^
Mexican American	57 (7.08%)	41 (19.0%)	170 (12.7%)	
Other Hispanic	41 (5.09%)	14 (6.48%)	118 (8.82%)	
Non-Hispanic white	368 (45.7%)	59 (27.3%)	611 (45.7%)	
Non-Hispanic black	233 (28.9%)	64 (29.6%)	256 (19.1%)	
Other Race—including multi-racial	106 (13.2%)	38 (17.6%)	183 (13.7%)	
**Weight (kg)**	82.7 (24.5)	86.6 (21.6)	87.4 (23.0)	^***^
**Height (cm)**	169 (9.31)	170 (9.65)	168 (9.43)	^*^
**BMI (kg/m** ^**2**^ **)**	28.8 (8.11)	30.0 (6.86)	30.8 (7.39)	^***^
**Marital status**				^***^
Married or living with partner	397 (49.9%)	105 (50.0%)	809 (60.9%)	
Never married	184 (23.1%)	54 (25.7%)	135 (10.2%)	
Widowed, divorced, or separated	215 (27.0%)	51 (24.3%)	384 (28.9%)	
**Comorbidities**				
**High blood pressure**	292 (36.3%)	73 (33.8%)	669 (50.2%)	^***^
**High cholesterol levels**	215 (26.9%)	66 (31.3%)	635 (47.9%)	^***^
**Diabetes**				^***^
Yes	96 (11.9%)	27 (12.5%)	313 (23.4%)	
No	695 (86.3%)	183 (84.7%)	975 (72.9%)	
Borderline	14 (1.74%)	6 (2.78%)	49 (3.66%)	
**Failing kidneys**	19 (2.39%)	8 (3.81%)	91 (6.86%)	^***^
**Anemia**	31 (3.86%)	6 (2.79%)	70 (5.25%)	0.14
**Congestive heart failure**	27 (3.41%)	5 (2.38%)	101 (7.64%)	^***^
**Coronary heart disease**	29 (3.66%)	9 (4.29%)	123 (9.30%)	^***^
**Stroke**	59 (7.44%)	8 (3.81%)	93 (7.02%)	0.17
**Chronic obstructive pulmonary disease**	107 (13.5%)	13 (6.19%)	128 (9.66%)	^**^
**Malignancy**	69 (8.68%)	18 (8.57%)	214 (16.1%)	^***^
**Renal function**				
Blood Urea Nitrogen (mmol/L)	4.83 (1.83)	4.93 (1.84)	6.05 (2.60)	^***^
Creatinine, refrigerated serum (umol/L)	79.1 (23.1)	79.2 (21.9)	88.1 (45.0)	^***^
eGFR (ml/min/1.73 m^2^)	96.5 (21.9)	97.5 (21.7)	84.1 (24.6)	^***^
Uric acid (umol/L)	321 (88.6)	325 (81.9)	347 (92.6)	^***^
Albumin creatinine ratio (mg/g)	37.1 (162)	34.7 (168)	58.4 (283)	0.11
**Metabolites of nicotine**				
Serum cotinine (ng/mL)	263 (139)	124 (140)	35.8 (118)	^***^
Serum hydrocotinine (ng/mL)	102 (76.9)	49.7 (57.6)	16.0 (61.6)	^***^

* *p < 0.05*,

** *p < 0.01*,

*** *p < 0.001 using statistical methods of ANOVA and Chi-squared test*.

The non-smoker group had worse indices of the renal function than the other two smoking groups, but no difference was found in albumin–creatinine ratio levels (Table 3). The cotinine and hydrocotinine levels were positively associated with smoking frequency (Table 3; Figure 1B). They were highly correlated with each other as well with the Pearson correlation r 0.85 (*p* < 0.001) (Figure 1C).

### Sleep Quality and Smoking Characteristics of the Study Population

Study population with sleep duration < 6 h tended to had worse sleep qualities of snoring, breath cessation, and having trouble in sleeping. This sleep subgroup was more likely to smoke every day, smoke at least 100 cigarettes in life, and smoke within 30 min after waking up (Table 4). Over half percent of nmSleep and moreSleep did not smoke recently. The average sleep duration of edSmoking, stSmoking, and noSmoking were 7.42 ± 1.91, 7.50 ± 1.77, and 7.66 ± 1.66 h, respectively. There was no significant difference in the sleep qualities among the three smoking subgroups (Table 5). The U-shaped effects of cotinine levels among the three sleep subgroups were only observed in the study population smoked sometimes that normal sleep duration group had lower cotinine levels (Figure 1D).

**Table 4:** Sleep quality and smoking characteristics of study population grouped by sleep duration.

	**Sleep duration on weekdays**			
	**≤6 h**	**6–9 h**	**≥9 h**	***p*-values**
	**(*n* = 1,118)**	**(*n* = 5,652)**	**(*n* = 1,499)**	
**Sleep quality**				
**Sleep duration (h)**	5.23 (0.969)	7.54 (0.617)	9.74 (1.01)	^***^
**How often do you snore**				^***^
Never	284 (27.9%)	925 (28.2%)	479 (34.2%)	
Rarely−1–2 nights a week	226 (22.2%)	828 (25.2%)	316 (22.5%)	
Occasionally−3–4 nights a week	185 (18.2%)	648 (19.7%)	235 (16.8%)	
Frequently−5 or more nights a week	324 (31.8%)	881 (26.8%)	372 (26.5%)	
**How often do you snore or have breath cessation**				^***^
Never	778 (74.0%)	2,592 (77.6%)	1,104 (77.7%)	
Rarely−1–2 nights a week	133 (12.7%)	408 (12.2%)	158 (11.1%)	
Occasionally−3–4 nights a week	59 (5.61%)	200 (5.98%)	102 (7.18%)	
Frequently−5 or more nights a week	81 (7.71%)	142 (4.25%)	57 (4.01%)	
**Having trouble sleeping**	346 (30.9%)	858 (24.2%)	417 (27.9%)	^***^
**Smoking status**				
**Smoked at least 100 cigarettes in life (yes/no)**	489 (45.3%)	1,293 (38.4%)	577 (40.8%)	^***^
**Age started smoking cigarettes regularly (years)**	17.8 (6.06)	18.0 (6.53)	17.9 (6.33)	0.89
**Do you now smoke cigarettes**				^**^
Every day	202 (41.3%)	417 (32.3%)	186 (32.2%)	
Some days	50 (10.2%)	120 (9.28%)	46 (7.97%)	
Not at all	237 (48.5%)	756 (58.5%)	345 (59.8%)	
**How soon after waking do you smoke**				^*^
≤ 5 min	54 (27.7%)	85 (20.3%)	56 (31.3%)	
6 ~ 30 min	66 (33.8%)	157 (37.5%)	48 (26.8%)	
≥30 min	75 (38.5%)	177 (42.2%)	75 (41.9%)	
**# days smoked cigarettes during past 30 days**	25.6 (8.65)	24.2 (10.0)	24.8 (9.19)	0.16
**Average # cigarettes/day during past 30 days**	11.2 (8.06)	11.2 (8.77)	10.3 (7.53)	0.42
**Tried to quit smoking (yes/no)**	126 (49.6%)	290 (53.0%)	125 (53.4%)	0.62

* *p < 0.05*,

** *p < 0.01*,

*** *p < 0.001 using statistical methods of ANOVA and Chi-squared test*.

**Table 5:** Sleep and smoking characteristics of study population grouped by smoking.

	**Do you now smoking?**			
	**Every day**	**Some days**	**Not at all**	***p*-values**
	**(*n* = 805)**	**(*n* = 216)**	**(*n* = 1,338)**	
**Sleep quality**				
**Sleep duration (h)**	7.42 (1.91)	7.50 (1.77)	7.66 (1.66)	^*^
**How often do you snore**				0.14
Never	203 (27.5%)	53 (27.0%)	277 (22.8%)	
Rarely−1–2 nights a week	144 (19.5%)	49 (25.0%)	274 (22.6%)	
Occasionally−3–4 nights a week	136 (18.4%)	31 (15.8%)	242 (19.9%)	
Frequently−5 or more nights a week	255 (34.6%)	63 (32.1%)	422 (34.7%)	
**How often do you snort or have breath cessation**				0.59
Never	516 (70.3%)	159 (77.6%)	874 (71.0%)	
Rarely−1–2 nights a week	107 (14.6%)	23 (11.2%)	174 (14.1%)	
Occasionally−3–4 nights a week	61 (8.31%)	12 (5.85%)	95 (7.72%)	
Frequently−5 or more nights a week	50 (6.81%)	11 (5.37%)	88 (7.15%)	
**Having trouble sleeping**	296 (36.8%)	66 (30.6%)	464 (34.7%)	0.21
**Smoking status**				
**Age started smoking cigarettes regularly (years)**	17.9 (6.07)	18.9 (9.28)	17.8 (5.97)	0.07
**How soon after waking do you smoke**				
≤ 5 min	194 (24.9%)	–	–	
6 ~ 30 min	270 (34.7%)	–	–	
≥30 min	315 (40.4%)	–	–	
**# days smoked cigarettes during past 30 days**	29.4 (2.99)	11.3 (7.15)	–	^***^
**Average # cigarettes/day during past 30 days**	12.8 (8.07)	4.13 (4.99)	–	^***^

* *p < 0.05*,

*** *p < 0.001 using statistical methods of ANOVA and Chi-squared test*.

### The Interactive Impacts of Sleep Duration and Smoking on the Renal Function

**Figure 2:**
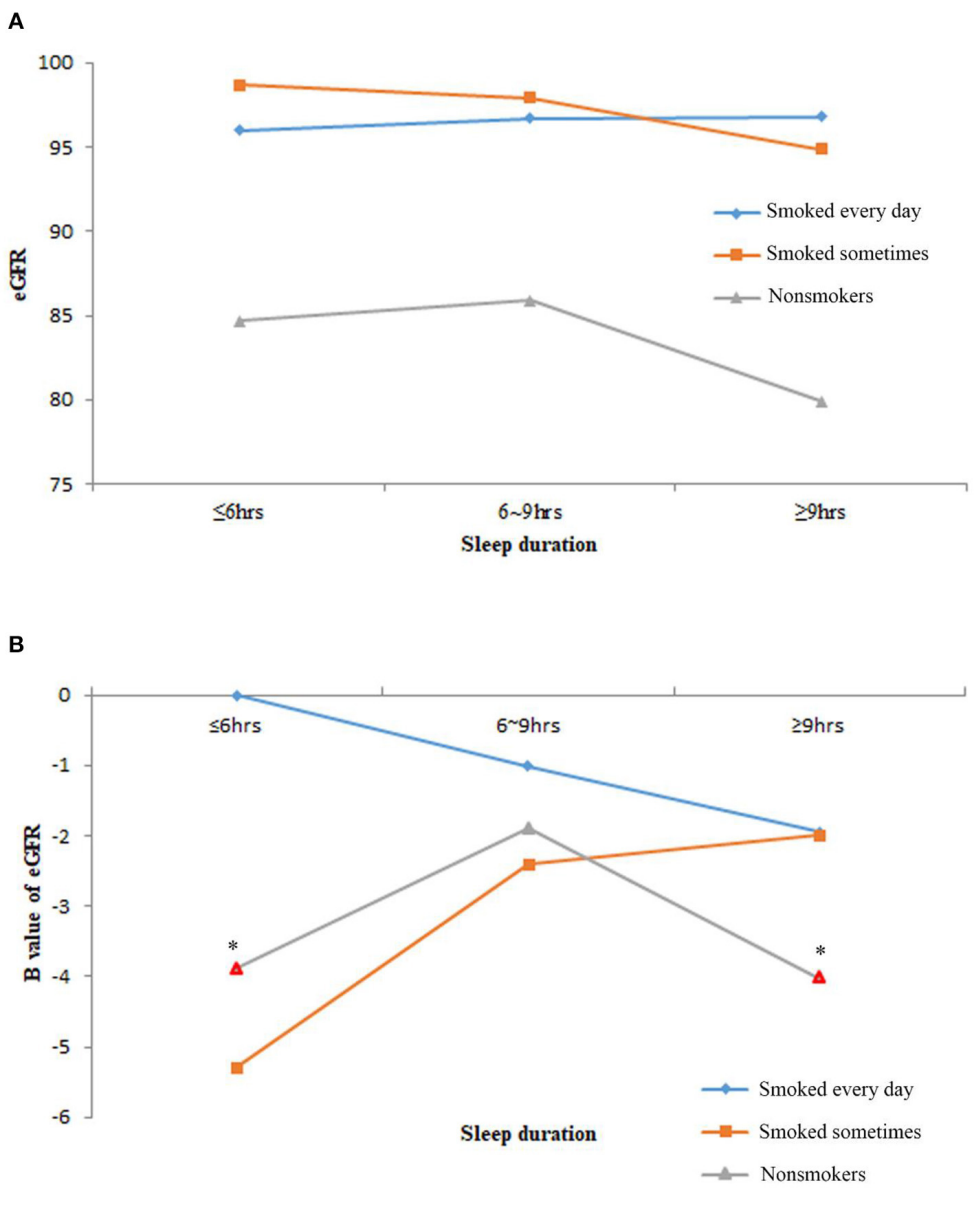
The estimated glomerular filtration rate (eGFR) levels of the nine subgroups of study population by sleep duration and smoking frequency. **(A)** The average eGFR levels of nine subgroups. **(B)** The B value of eGFR in multivariable linear regression models after adjusting age, race, body mass index (BMI), marital status, frequency of snoring or breath cessation, diabetes, high blood pressure, high cholesterol, anemia, congestive heart failure, coronary heart disease and stroke. Marks in red border and asterisks denoted *p* < 0.05 in the multivariable linear regression models.

The baseline eGFR levels of the study population who smoked every day or were sometimes similar and higher than the non-smokers (Figure 2A). Although the interaction term of smoking and sleep duration was not significant in the multivariable linear regression analysis (data not shown), the patterns of eGFR levels in the nine subgroups varied. For the study population who smoked every day, the longer the sleep time, the lower the eGFR levels. For non-smokers, the reverse U-shaped effects of eGFR levels were observed. Non-smokers with normal sleep duration had higher eGFR levels as compared with the other two sleep groups. For the study population who smoked sometimes, the longer sleep duration, the higher were the eGFR levels (Figure 2B).

**Figure 3:**
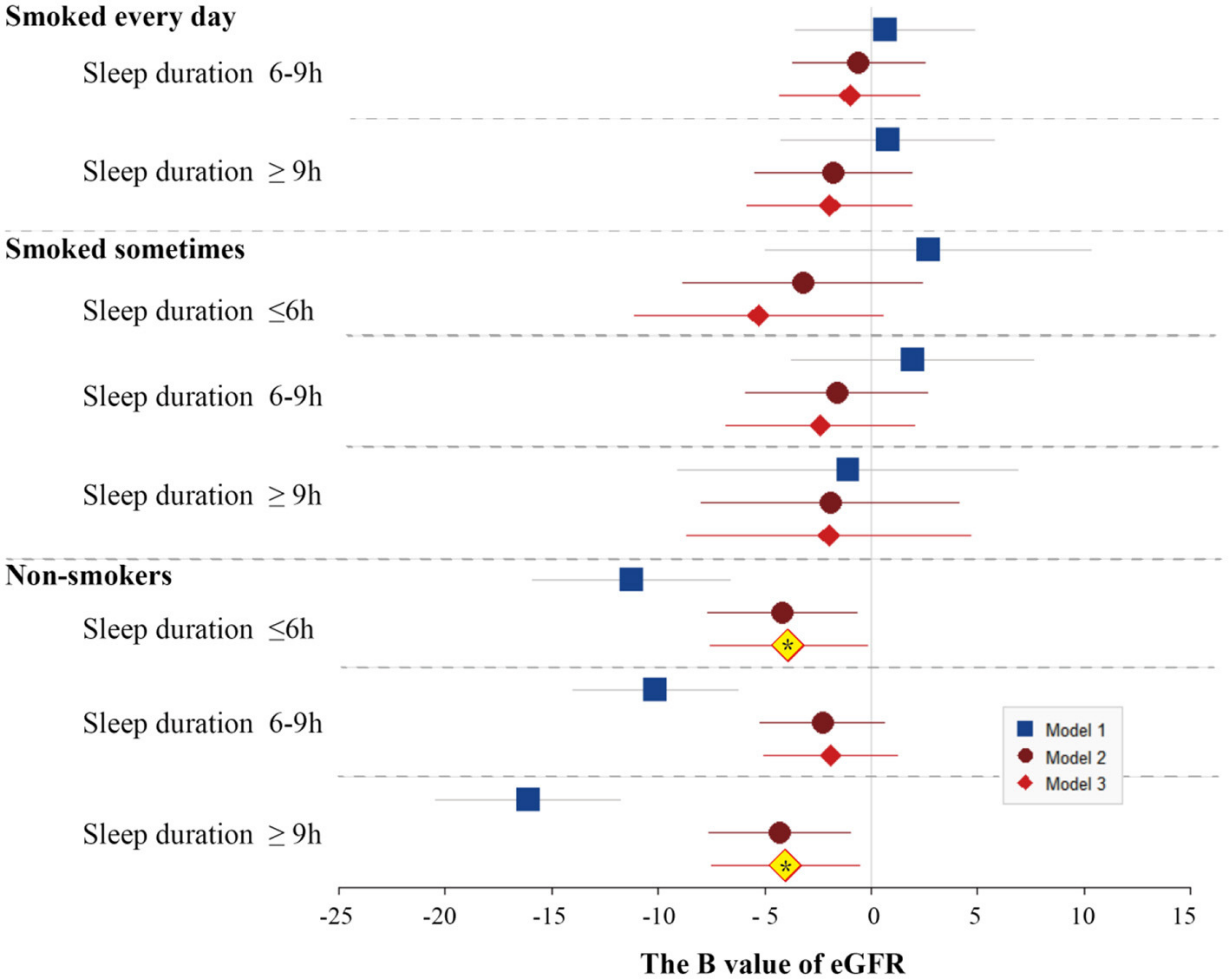
Forest plots of B values of eGFR in the univariable and multivariable linear regression models among study population subgroups categorized by smoking and sleep duration. The subgroup of longer sleep duration and smoking every day was served as the reference group in the models. Model 1 was not adjusted. Model 2 was adjusted by age, race, BMI, and marital status. Model 3 was adjusted by age, race, BMI, marital status, frequency of snoring or breath cessation, diabetes, high blood pressure, high cholesterol, anemia, congestive heart failure, coronary heart disease, and stroke.

After the adjustment of the demographic characteristics (age, race, BMI, and marital status), sleep quality (snoring or breathing cessation), and comorbidities (diabetes, high blood pressure, high cholesterol, anemia, congestive heart failure, coronary heart disease, and stroke), only noSmoking-lessSleep and noSmoking-moreSleep had significantly lowered eGFR levels compared with edSmoking-lessSleep. The U-shaped effect of sleep duration on the renal function was significantly observed in noSmoking that nmSleep had better eGFR than the other two sleep groups (Figure 3). Normal sleep duration was a predominant profactor of the renal function in noSmoking. For the smokers, sleep duration had no significant effect on eGFR. A controversial finding is that noSmoking–moreSleep or noSmoking–lessSleep had lower eGFR than edSmoking–lessSleep in both the univariable and multivariable models (Figure 3).

## Discussion

Accumulating the clinical evidence suggests that cigarette smoking has a negative effect on the renal function, kidney dimensions (18), and CKD development of different etiologies, including DM, and hypertension (19, 20). Cigarette smoking is one of the most important modifiable renal risk factors (21). Nicotine, a major tobacco alkaloid, associates smoking with renal dysfunction (22, 23). The risk of adverse kidney outcomes was incrementally higher as a smoking pack–years increased (24). Exposure to nicotine has been strongly shown to enhance renal oxidative stress (22) and kidney failure (25). Chronic exposure to nicotine accelerates the onset and progression of renal diseases in habitual cigarette smokers.

A major pathway of nicotine metabolism is C-oxidation, followed by cotinine; and the subsequent hydroxylation to trans-3′-hydroxycotinine. Moreover, 85–95% of the total nicotine uptake is eliminated as cotinine, hydroxycotinine, and glucuronides in the urine (26, 27). Cotinine has a longer plasma half-life than nicotine and showed a dose-dependent effect of smoking exposure (26, 28–30). This is in line with our finding; smoking frequency and serum cotinine and hydrocotinine levels were positively associated with each other. Therefore, we divided the smoking groups by the self-report of smoking frequency from the NHANES dataset.

The mechanisms of smoking-related renal damage are poorly understood, but the damage is likely caused by vascular and tubular effects (22). Smoking may sensitize the kidney to ischemic insults and perhaps facilitate the progression of acute kidney injury to chronic kidney injury (22). Nicotine increases the severity of renal injury in animal models leading to acute kidney injury, DM, acute nephritis, and subtotal nephrectomy (19). Nicotine stimulates the proliferation and hypertrophy of mesangial cells. Nicotine administration to sham rats increased total proteinuria but not albuminuria, indicating that nicotine directly affects tubular protein reabsorption (31). In humans, nicotine induces transitory increases in blood pressure accompanied by reductions in eGFR and effective renal plasma flow (19).

We found that the serum blood urea nitrogen and creatinine were higher and that eGFR levels were lower in the group of noSmoking than in the group of edSmoking and stSmoking after adjusting for age, race, BMI, marital status, and comorbidities (DM, hypertension, high cholesterol level, congestive heart failure, coronary heart disease, angina/angina pectoris, heart attack, stroke, emphysema, chronic bronchitis, and anemia). This finding is contradictory to the results of most studies (19–25).

In a study involving 28,409 individuals, smokers exhibited a slightly higher creatinine clearance rate than non-smokers at least in men after adjusting for hypertension (32). Moreover, the administration of nicotine to adolescent mice for 4 weeks incited higher oxidative stress and tubular injury than in adult kidney, but it did not modify creatinine levels (33). Does this higher creatinine clearance in smokers signify a better renal function? This increase may reflect a direct effect of smoking on tubular creatinine secretion or interfere with the estimation methods of creatinine. A 24-h urine collection would considerably eliminate any interference between smoking and estimation of creatinine levels (32). The creatinine-based eGFR raises as the smoking amount increases, whereas the cystatin C-based eGFR decreases (34). This finding might indicate that creatinine-based eGFR, which was adopted in this study, may not be an ideal marker to estimate the relationship between smoking and renal function. Current smoking status cannot reflect the history of past exposure to cigarettes, and nicotine tends to be a short-term exposure marker of smoking. This is a limitation of this study. A high proportion of noSmoking individuals may have had a history of long past exposure to smoking cigarettes.

The finding of better renal function in smokers might have some biological plausibility. Lower doses of subacute nicotine administration can enhance renal function (35). Moreover, nicotine has a protective effect against neurotoxic insults and may be of potential therapeutic value in Parkinson's disease (36). In addition, cotinine reduced fear memory and anxiety after fear conditioning and improved working memory in a mouse model of Alzheimer's disease and in a monkey model of schizophrenia (37). Nicotine pretreatment reduced tubular damage (tubular cell apoptosis and proliferative response) due to an innate immune response in animal model experiments (38).

We found that the normal sleep duration of 6–9 h is associated with better eGFR and other renal function indices (blood urea nitrogen, creatinine, and uric acid) compared with the other two sleep duration groups; however, there was no difference in the albumin–creatinine ratio. In agreement with the previous studies, short and long sleep durations have been associated with adverse health outcomes in the general population (39, 40) and in patients with CKDs and diabetic kidney disease (DKD) (39, 41, 42). Overall, the cutoffs of normal sleep duration (6–8 or 6–9 h) may differ slightly, but the finding remained consistent (42). Physiological evidence indicated that sleep influences kidney function. The genetic risk score for short, but not long, sleep duration was significantly related with a higher risk of CKD stages 3–5 (6).

A poor sleep profile or quality is another important risk factor of increasing CKDs risk (42). SA is a condition that has serious health consequences, has an increased risk of death, and is common in patients with CKDs (43, 44) and DKD (45). Obstructive SA-related hypoxia causes several negative systemic effects, including oxidative stress (46, 47), inflammation, and sympathetic activation, all of which contribute to the progression of renal disease. In turn, CKD can result in the increased severity of SA by inducing uremic neuropathy and myopathy, altered chemosensitivity, and hypervolemia (43).

Sleeping behaviors and smoking have a reciprocal effect and a moderate correlation with genetics (48). Severe smoking status appears to have a causal effect on the circadian rhythm, and some evidence has shown that insomnia increases smoking heaviness and impedes cessation (48). Indeed, cigarette smoking has been shown to be associated with sleep disturbance *via* prolonged sleep-onset latency, higher dopamine levels, and lower dopamine transporter levels in the cerebrospinal fluid of active smokers (49). The symptoms of cigarette smoking and nicotine dependence were associated with poor sleep quality in young adult smokers (50). Both oral nicotine administration and abstinence led to sleep disturbances in mice (51). In line with these findings, lessSleep individuals were shown to be more likely to smoke and had higher levels of cotinine and hydrocotinine in this study.

Many studies have discussed the individual association of sleep and smoking with the renal function in general, CKDs or DKD population. To the best of our knowledge, this is the first study to discuss the interaction effect of sleep duration and smoking on renal function. We observed that the non-smokers who had less or more sleep exhibited significantly lower eGFR levels compared with those who smoked every day and slept less. This association was independent of demographic characteristics (age, race, BMI, marital status), sleep quality of snoring or breathing cessation, and comorbidities (high blood pressure, high cholesterol, anemia, congestive heart failure, coronary heart disease, and stroke). This finding contradicts those of many studies that the risk of adverse kidney outcomes was incrementally higher as smoking pack–years increased (18, 19, 24). Even, we evaluated the eGFR of normal or abnormal serum cotinine groups, the average eGFR levels of the abnormal cotinine group were still slightly higher than the normal cotinine group. In noSmoking, the eGFR levels of the study population with normal levels of cotinine or hydrocotinine were lower than or equal to those with abnormal levels of cotinine.

The contradictory findings of smoking and nicotine on adverse kidney outcomes can be attributed to the following factors: (1) information on the nicotine exposure history of dose and length are not available (52), (2) creatinine-based eGFR may not be an ideal marker to estimate the relationship between smoking and the renal function (34), (3) other harmful ingredients from cigarettes have a greater effect on the renal function than nicotine (53), and (4) the non-smoking group possessed more risk factors (older age, male predominance, BMI, higher proportion of non-Hispanic white race, and higher prevalence of comorbidities). Despite our efforts to adjust for the potential confounders in the models, other residual confounders may still exist. During modeling, we found that after adjusting for age, the difference in eGFR levels between the smokers and non-smokers decreased. Older age played an important role in the development of adverse kidney function.

Among the non-smokers, sleep duration had a significant effect on eGFR. Either less or more sleep duration was harmful to the renal function, which was associated with a decline in eGFR. Normal sleep duration is an important profactor of the renal function in the non-smoking population. No statistical significance of interaction effect was found between sleep duration and smoking status on eGFR in the multivariable linear regression models. However, the effects of sleep duration on eGFR levels varied with the smoking frequency. The eGFR levels of edSmoking increased as the sleep duration decreased, whereas the eGFR levels of stSmoking increased as the sleep duration increased. The U-shape effects of eGFR levels were observed among the non-smokers; the group with normal sleep duration had the highest eGFR levels. To the best of our knowledge, this is the first study to examine the interaction of sleep duration and smoking status on eGFR. However, because the NHANES datasets are cross-sectional, we were unable to understand the causal effects. Moreover, a reciprocal and prospective relationship exists between smoking and sleeping problems (12), and further research is required to unravel whether renal function has a reciprocal effect on sleep as well.

## Conclusions

The effects of sleep duration on the renal function varied with smoking frequency. The non-smokers with short or long sleep duration exhibited significantly lower eGFR levels compared with those who smoked every day and slept less after adjusting for demographic characteristics, sleep quality, and comorbidities. Normal sleep duration was a protective and more crucial factor for the non-smokers than for smokers. As this was a cross-sectional study, further longitudinal studies are required to confirm the causal effects of sleep and smoking on the renal function.

## Data Availability Statement

Publicly available datasets were analyzed in this study. This data can be found at: National Health and Nutrition Examination Survey https://www.cdc.gov/nchs/nhanes/index.htm.

## Author Contributions

Y-CL and Y-TC: data curation. Y-TC: formal analysis, software, and writing the original draft. H-EW and C-CW: resources. C-CW: supervision. H-EW, C-CW, Y-CL, C-MZ, PC, K-CL, and C-MC: writing, reviewing, and editing. All authors contributed to the article and approved the submitted version.

## Funding

This study was supported by research grants from the Tri-Service General Hospital (TSGH-C05-110033) and the Ministry of Science and Technology (MOST110-2314-B-016-014), Taiwan.

## Conflict of Interest

The authors declare that the research was conducted in the absence of any commercial or financial relationships that could be construed as a potential conflict of interest.

## Publisher's Note

All claims expressed in this article are solely those of the authors and do not necessarily represent those of their affiliated organizations, or those of the publisher, the editors and the reviewers. Any product that may be evaluated in this article, or claim that may be made by its manufacturer, is not guaranteed or endorsed by the publisher.
